# Defining the Conformational Features of Anchorless, Poorly Neuroinvasive Prions

**DOI:** 10.1371/journal.ppat.1003280

**Published:** 2013-04-18

**Authors:** Cyrus Bett, Tim D. Kurt, Melanie Lucero, Margarita Trejo, Annemieke J. Rozemuller, Qingzhong Kong, K. Peter R. Nilsson, Eliezer Masliah, Michael B. Oldstone, Christina J. Sigurdson

**Affiliations:** 1 Department of Pathology, University of California, San Diego, La Jolla, California, United States of America; 2 Department of Neuroscience, University of California, San Diego, La Jolla, California, United States of America; 3 Dutch Surveillance Centre for Prion Diseases, University Medical Centre Utrecht, Utrecht, The Netherlands; 4 Department of Pathology, Case Western Reserve University, Cleveland, Ohio, United States of America; 5 Department of Chemistry, Biology, and Physics, Linkoping University, Linkoping, Sweden; 6 Department of Immunology and Microbial Science, The Scripps Research Institute, La Jolla, California, United States of America; 7 Department of Pathology, Immunology, and Microbiology, University of California, Davis, Davis, California, United States of America; Dartmouth Medical School, USA, United States of America

## Abstract

Infectious prions cause diverse clinical signs and form an extraordinary range of structures, from amorphous aggregates to fibrils. How the conformation of a prion dictates the disease phenotype remains unclear. Mice expressing GPI-anchorless or GPI-anchored prion protein exposed to the same infectious prion develop fibrillar or nonfibrillar aggregates, respectively, and show a striking divergence in the disease pathogenesis. To better understand how a prion's physical properties govern the pathogenesis, infectious anchorless prions were passaged in mice expressing anchorless prion protein and the resulting prions were biochemically characterized. Serial passage of anchorless prions led to a significant decrease in the incubation period to terminal disease and altered the biochemical properties, consistent with a transmission barrier effect. After an intraperitoneal exposure, anchorless prions were only weakly neuroinvasive, as prion plaques rarely occurred in the brain yet were abundant in extracerebral sites such as heart and adipose tissue. Anchorless prions consistently showed very high stability in chaotropes or when heated in SDS, and were highly resistant to enzyme digestion. Consistent with the results in mice, anchorless prions from a human patient were also highly stable in chaotropes. These findings reveal that anchorless prions consist of fibrillar and highly stable conformers. The additional finding from our group and others that both anchorless and anchored prion fibrils are poorly neuroinvasive strengthens the hypothesis that a fibrillar prion structure impedes efficient CNS invasion.

## Author Summary

Prions cause fatal neurodegenerative disease in humans and animals and there is currently no treatment available. The cellular prion protein is normally tethered to the outer leaflet of the plasma membrane by a glycophosphatidyl inositol (GPI) anchor. A rare stop codon mutation in the *PRNP* gene leads to the production of GPI-anchorless prion protein and the development of familial prion disease, which has been reproduced in mouse models. GPI-anchorless prions in humans or mice form large, dense plaques containing fibrils in the brain that vary from the more common non-fibrillar prion aggregates. Here we investigated the biochemical differences between GPI-anchored and GPI-anchorless prions. We also assessed the capacity of GPI-anchorless prions to spread from entry sites into the central nervous system. We found that infectious GPI-anchorless prions were extraordinarily stable when exposed to protein denaturing conditions. Additionally, we show that GPI-anchorless prions rarely invade the central nervous system and then only after long incubation periods, despite their presence in extraneural tissues including adipose tissue and heart. Our study shows that GPI-anchored prions converted into GPI-anchorless prions become extraordinarily stable, more resistant to enzyme digestion, and are poorly able to invade the nervous system.

## Introduction

Prions are pathogenic protein aggregates that cause progressive neurodegenerative disease in humans and animals [1], [2]. Most infectious prions spread from peripheral entry sites into the central nervous system (CNS), and can also spread from the CNS to extracerebral sites, such as muscle [3], [4]. This ability of prions to shuttle in and out of the CNS is extraordinary and rare among aggregated proteins or amyloids. Among prions, there is variable capacity to spread to the CNS, a process termed neuroinvasion [5].

A cardinal feature of prion disease is the deposition of PrP^Sc^, a multimer of misfolded prion protein that templates the structural conversion of the host-encoded monomer, PrP^C^, in an autocatalytic process [6]. Intriguingly, distinct infectious prion strains, which have the same amino acid sequence, show dramatic differences in the disease incubation period and brain regions targeted [7], [8], [9]. Among prion strains, there can be remarkable heterogeneity in the biochemical and physical properties of PrP^Sc^. For example, PrP^Sc^ may vary in aggregate size [10], stability in chaotropes [11], glycoform profile [12], [13], [14], and resistance to enzymatic degradation [15]. Thus, biochemical differences among prion strains seem to be due to distinct structural arrangements of PrP^Sc^ [16], [17], [18].

The processed PrP^C^ glycoprotein is composed of approximately 210 residues and is tethered to the plasma membrane by a glycosylphosphatidyl inositol (GPI) anchor [19]. The GPI-anchor has been shown to be dispensable for prion conversion in vitro [20]. Indeed, individuals expressing C-terminally truncated or full length PrP^C^ lacking the GPI anchor are at risk for developing familial prion disease [Gerstmann-Sträussler-Scheinker disease (GSS)] and amyloid plaques in the brain [21]. A recent report shows that transgenic mice overexpressing GPI-anchorless PrP^C^ also form plaques spontaneously [22].

Exposure of anchorless PrP-expressing mice to RML prions leads to extensive cerebral angiocentric amyloid plaques, which is a striking morphologic switch from the diffuse, granular prion aggregates seen with the same RML prions in wild type mice [23], [24]. GPI-anchorless prions form fibrils [24], which may not be solely due to the lack of the GPI anchor, as the anchorless prions are also underglycosylated and extracellular. These anchorless fibrillar prions have been shown to be poorly neuroinvasive after an extensive series of different peripheral routes of exposure [25]. We and others have previously identified GPI-anchored fibrillar prions that are poorly neuroinvasive, which is in contrast to the rapid neuroinvasion typical of nonfibrillar strains [26], [27], [28]. We found that fibrillar prions were highly stable in chaotropes as compared to the nonfibrillar strains [26].

To better understand how the GPI-anchor and fibrillar structure impact the ability of a prion to spread to the CNS, we performed a comprehensive analysis of the biophysical properties of GPI-anchorless prions and correlated our findings with the disease phenotype. To then assess whether the physical properties of anchorless prions in mice applied to natural disease, we measured the chaotrope stability of anchorless prions from the brain of a patient with a rare familial prion disease due to a *PRNP* mutation coding for Q227X.

## Results

### Serial passage of anchorless-RML prions leads to a shortening of the incubation period

Transgenic GPI-anchorless mice [Tg(GPI^−^PrP)] express unglycosylated and monoglycosylated PrP at approximately 0.5-fold wild type (WT) mouse levels [23]. Tg(GPI^−^PrP) mice inoculated intracerebrally (IC) with mouse-adapted RML prions develop terminal prion disease after an extended incubation period of >300 days post-inoculation (dpi) [24]. To assess whether anchored prions inefficiently convert anchorless PrP^C^ due to a transmission barrier, we serially passaged GPI^−^RML in the Tg(GPI^−^PrP) mice. Indeed, the incubation period decreased to 198±7 days on second passage, and was similar on third passage (205±12 days) (Table 1), suggestive of a transmission barrier caused by the GPI-anchor. PrP^Sc^ levels were only slightly higher in passage 2 (Figure S1). Clinical signs were equivalent among passages in the Tg(GPI^−^PrP) mice and consisted of inactivity, weight loss, ataxia, tremors, stiff tail, and kyphosis.

**Table 1:** Prion incubation period in mice that express GPI-anchored or anchorless PrP^C^.

Mouse PrP^C^	Prion strain	Inoculation route	Incubation period (days post-inoculation)^1^
GPI^+^PrP	RML	IC	163±4
GPI^+^PrP	RML	IP	227±1
GPI^+^PrP	GPI^−^RML	IC	156±1
GPI^−^PrP	GPI^−^RML, 2^nd^ psg	IC	198±7
GPI^−^PrP	GPI^−^RML-3^rd^ psg	IC	205±12
GPI^−^PrP	GPI^−^RML	IP	454±23
GPI^+^PrP	22L	IC	141±2
GPI^−^PrP	GPI^−^22L, 2^nd^ psg	IC	324±18

1 Mean ± SE in days from inoculation to terminal prion disease.

**Figure 1:**
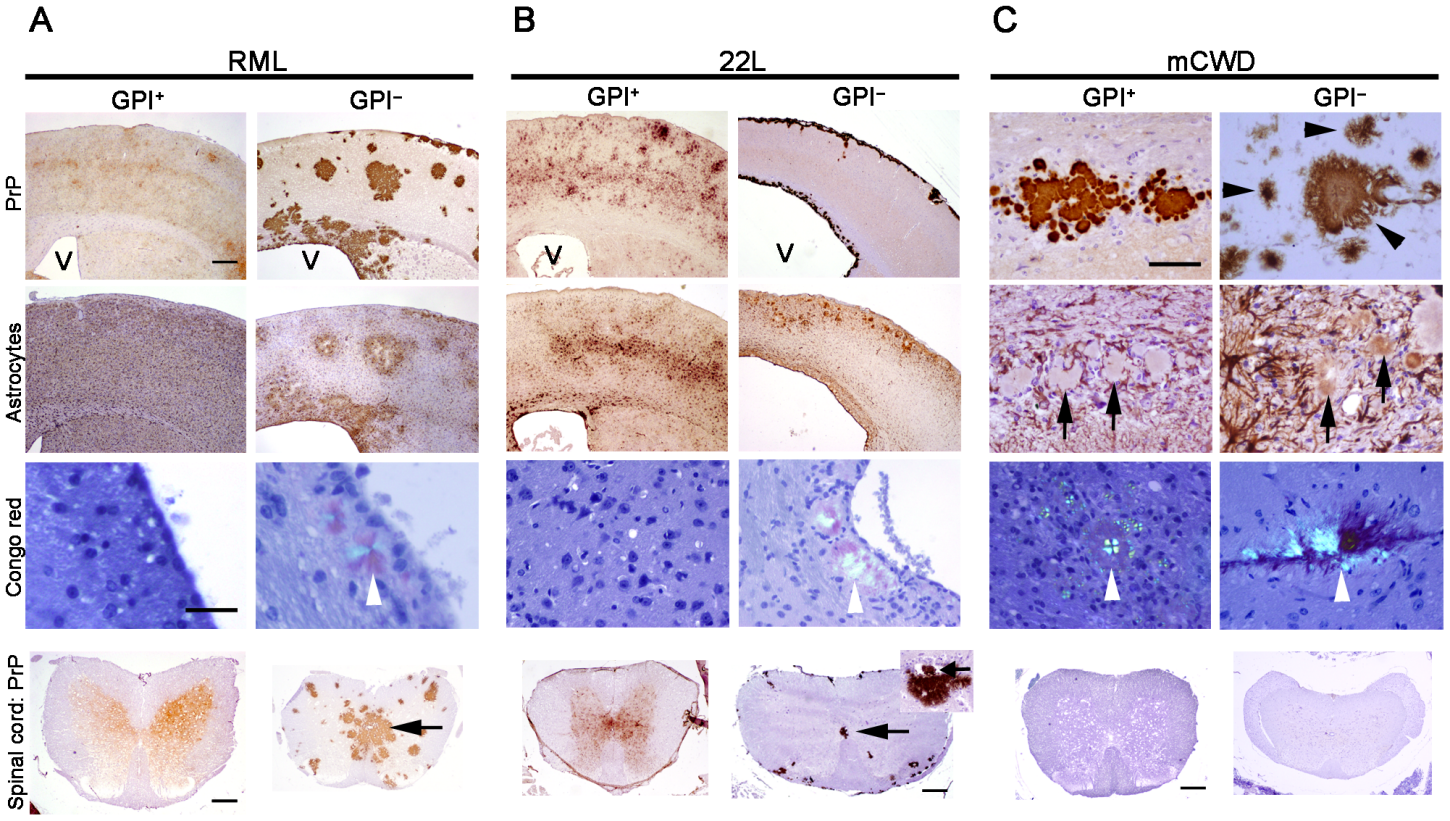
Comparison of PrP aggregates and gliosis in the brains and spinal cords of prion-infected mice expressing anchored or anchorless PrP^C^. (A,B) RML and 22L aggregates appear as small, fine clusters that are diffusely distributed throughout affected brain regions, whereas the serially passaged anchorless prions consist of multifocal, extensive dense plaques. Note the distended ventricle (V) in the GPI^–^22L infected mouse consistent with hydrocephalus (B). Congo red binds only to the GPI^–^RML and GPI^–^22L plaques (arrowhead). Anchorless plaques in the spinal cord are concentrated around the central canal (arrow) as well as in the white matter. Inset of GPI^–^22L shows a plaque within the central canal (arrow). (C) mCWD prions in mice expressing anchored or anchorless PrP consist of dense large plaques that bind Congo red (arrowhead), but plaques showed fibrillar margins in the anchorless mice (arrowhead). Astrocytes are present primarily around plaques of all congophilic prions, yet are more diffusely activated in mice infected with noncongophilic prions. Scale bars = 500 µm (A and B, PrP and astrocytes), 100 µm (C, PrP and astrocytes), and 100 µm (Congo red). Scale bars for spinal cord = 500 µm, (GPI^+^RML, GPI^−^RML, GPI^+^22L, GPI^−^mCWD), 1 mm (GPI^−^22L), and 200 µm (GPI^+^mCWD).

**Figure 2:**
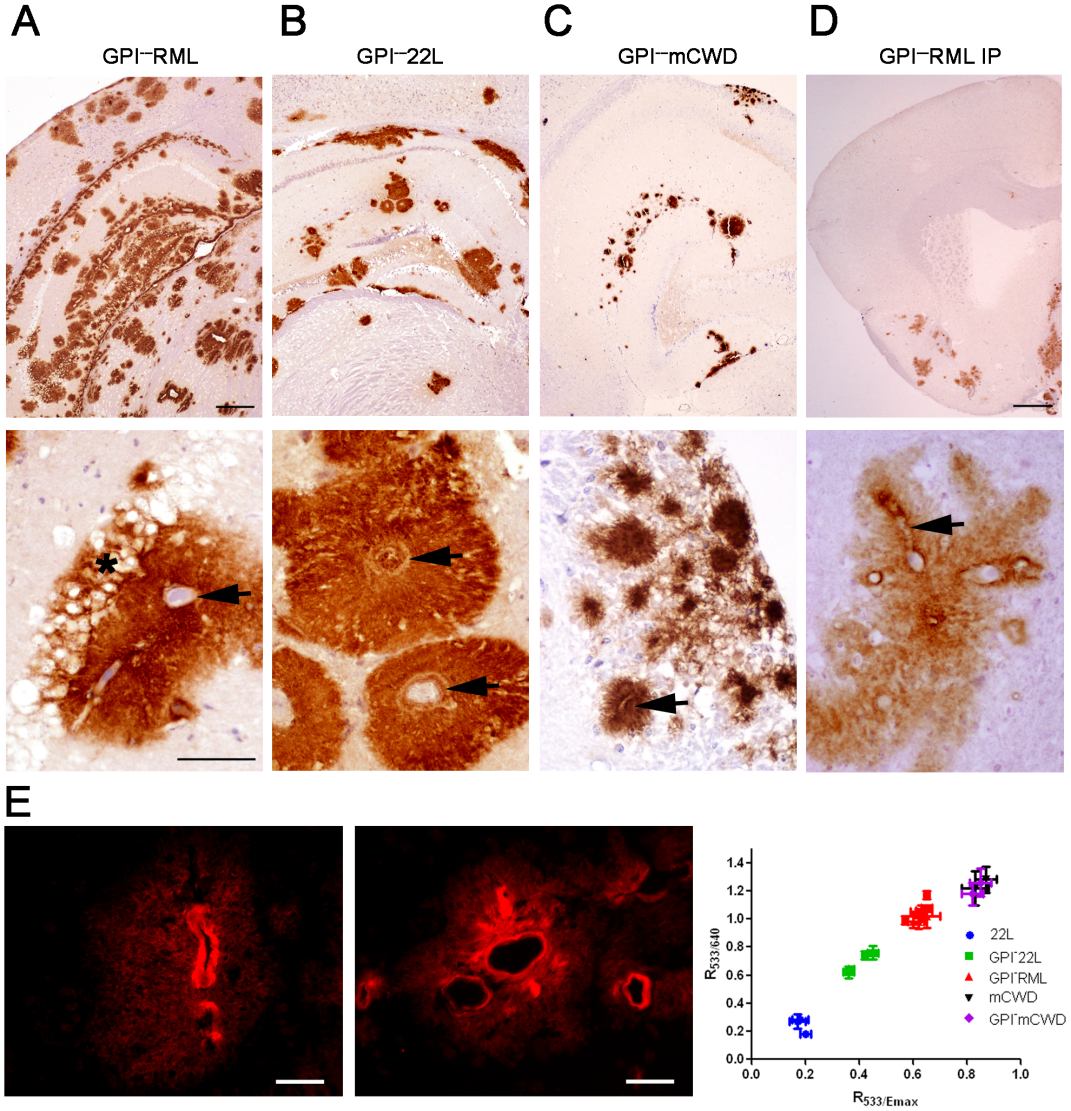
Distribution and morphology of GPI-anchorless plaques. (A and B) Serially passaged anchorless RML and 22L prions are widely scattered throughout the brain, predominantly around vessels (arrows), and occasionally obstructing the vessel (B, upper arrow). Shown here are cerebral cortex, hippocampus, and thalamus. Note how the hippocampal neurons are not replaced by the plaque (panel A, labelled ‘*’). (C) Anchorless mCWD prions were present primarily in the hippocampus and in the meninges, also around vessels (arrow). (D) After IP inoculation of GPI^–^RML prions, anchorless-prions showed limited spread to the brain and plaques surrounded vessels (lower panel, arrow). Scale bars = 500 µm (top row, A–C), 1 mm (top row, panel D), and 100 µm (bottom row). (E) PTAA-labeled GPI^–^RML (left) and GPI^–^22L (right) plaques in brain cryosections. A correlation diagram shows the ratios of the emitted light intensity from PTAA bound to prion aggregates at 533 nm/640 nm and at 533 nm/emission maximum. Scale bar = 20 µm.

We assessed the histopathology during serial passage of anchorless prions, however there was no apparent alteration in the plaque morphology or astrocyte reaction. In all passages, anchorless PrP appeared as multifocal to coalescing 50–200 µm dense plaques of PrP^Sc^, which radiated extensively from vessels, consistent with previous reports [24]. The plaques differed profoundly from the diffuse, nonvascular PrP^Sc^ deposits seen in RML-infected WT brain (Figure 1A). GPI^−^RML plaques were widely distributed throughout the brain, including the cerebral cortex, basal ganglia, hippocampus, thalamus, cerebellum and brainstem (Figures 1A and 2A), and surrounded the central canal of the spinal cord (Figures 1A). Reactive astrocytes were largely limited to areas surrounding the GPI^−^RML plaques, yet were diffuse in the RML-infected WT brain (Figure 1A). Congo red stained the GPI^−^RML plaques, but not the WT-RML deposits (Figure 1A). Ultrastructurally, the brain from the third passage of GPI^−^RML showed extensive extracellular loose mats of short fibrils which were sometimes surrounded by dystrophic neurites (Figure S2), similar to reports of brain from the first passage of GPI^−^RML [23].

To evaluate the serial passage of another anchorless prion strain, we performed a second passage of anchorless 22L (GPI^−^22L). All mice developed terminal prion disease after 324±18 days. On gross examination of some mice, the brain cortices were atrophied and the ventricles appeared dilated (also visible in Figure 1B), consistent with hydrocephalus. Histologically, there were large dense plaques radiating from vessels in the brain (Figure 2B). Plaques were particularly extensive surrounding the ventricles and the central canal (Figure 1B). In some cases, the ependymal cells around the central canal were eroded and plaques were present within the canal (Figure 1B, inset), even completely obstructing the canal lumen in some segments. Although hydrocephalus from cerebral atrophy is often described in prion disease, in this case, an obstruction to CSF flow may have been the cause. The GPI^−^22L plaque morphology differed profoundly from the WT-22L deposits, which were diffuse and failed to bind Congo red (Figure 1B).

To assess how anchorless PrP^C^ is converted by a fibrillar congophilic aggregate, we inoculated mCWD prions into the Tg(GPI^−^PrP) mice. Here we found that the anchorless plaques remained dense and congophilic (Figure 1C), and the morphology was only slightly modified in the anchorless PrP-expressing mice (Figure 2C). Some plaques no longer showed a sharp dense border as seen for the mCWD, but instead the borders were indistinct (Figures 1C and 2C), suggesting that only a subtle change occurs with this congophilic prion in the anchorless PrP-expressing mice.

Tg(GPI^−^PrP) mice express PrP^C^ in peripheral organs, most highly in the heart, kidney and testis [23]. Tg(GPI^−^PrP) mice infected with RML have been reported to accumulate PrP^Sc^ in the heart, adipose tissue, and spleen [25], [29], [30]. Similarly, PrP^Sc^ deposits were present in the heart and adipose tissue of Tg(GPI^−^PrP) mice infected with the passaged GPI^−^RML and GPI^−^22L, and also with GPI^−^mCWD (Figure S3), indicating that fibrillar anchorless prions have an altered organ tropism compared to anchored prions.

### Polythiophene acetic acid (PTAA) distinguishes anchored and anchorless prion subtypes

We next labeled frozen sections of anchored and anchorless prion-infected brain with PTAA, an amyloid-binding molecule that fluoresces at different wavelengths depending on the amyloid bound (Figure 2D). The PTAA emission spectra from the anchorless 22L were more red-shifted than those of anchorless RML, suggesting of distinct conformations (Figure 2E). Anchorless 22L was also distinguishable from anchored 22L, which showed red-shifted PTAA emission spectra (Figure 2E). The granular prion aggregates seen for RML prions in wild type mice were not stained by PTAA. Hence, the prion aggregates from both of the anchorless strains were different from their WT counterparts. Interestingly, the spectra from the anchored and anchorless mCWD prions were indistinguishable, yet distinct from anchorless RML and 22L (Figure 2E).

### Limited CNS invasion of anchorless prions following a peripheral exposure

Previous reports indicate that intraperitoneal (IP) inoculation of RML into Tg(GPI^−^PrP) mice did not cause clinical prion disease, and PrP^Sc^ was detectable in the brain of only 3 of 12 mice by 600 dpi [25]. To determine whether passaged anchorless RML prions would be more neuroinvasive than anchored RML prions, we exposed Tg(GPI^−^) mice to anchorless-RML prions by the IP route. Two Tg(GPI^−^PrP) mice died and the remaining Tg(GPI^−^PrP) mice were euthanized due to clinical signs of weight loss and kyphosis after 454±23 days post-IP inoculation, which was approximately 130% longer than when the same anchorless-RML prions were administered by the IC route (198±7 days) (Table 1). By comparison, all WT mice inoculated IP with RML developed terminal prion disease after 227±1 day, which was only 39% longer than the IC route (163±4 days).

In contrast to the widespread distribution of GPI^–^RML deposits after IC exposure, IP exposed Tg(GPI^−^) mice showed only scattered plaques in the meninges, olfactory bulb, cerebral cortex, basal ganglia, and cerebellum (Figure 2D) in one mouse and rare plaques (<10) in 3 other mice. Only one of the five Tg(GPI^−^PrP) mice showed PrP^Sc^ in brain by ELISA and by western blotting (Figure S4). However, there was abundant PrP^Sc^ in the spleen by western blot (Figure S4) and in the heart and adipose tissue by IHC, indicating substantial peripheral PrP^Sc^ reservoirs (Figure S3).

### Anchorless prions are primarily proteinase K-resistant

**Figure 3:**
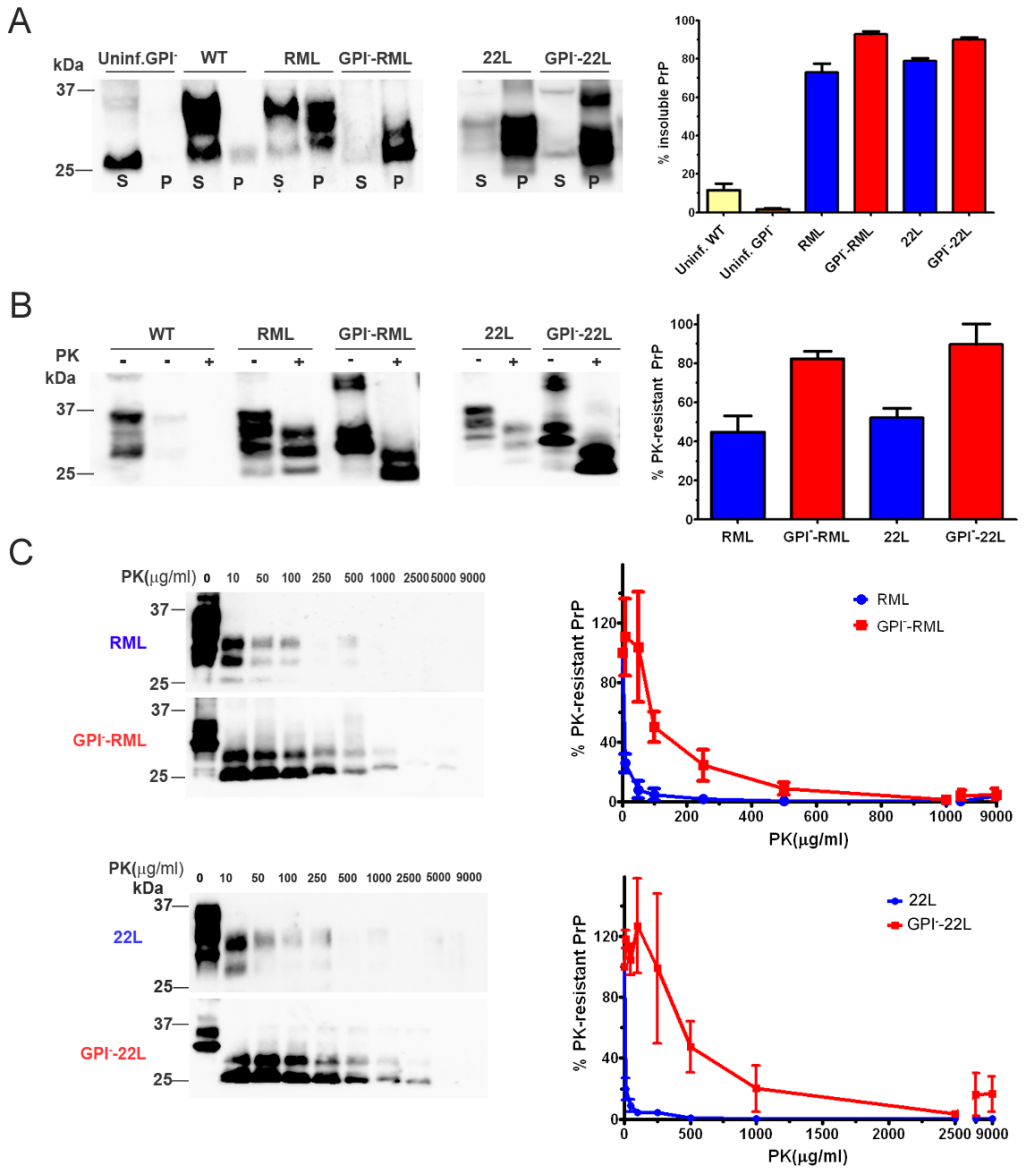
Solubility and PK-resistance of anchorless prions. (A) Soluble and insoluble PrP were measured in the anchored and anchorless RML and 22L infected brain samples by western blot. Graph shows the mean and SE from 3–4 mice per prion. (B) Insoluble PrP fraction: PrP levels were measured in undigested and PK digested samples. The graph shows the percentage of PK-resistant PrP over total insoluble PrP (mean and SE from 4 mice per prion). (C) PK-resistance of anchored and anchorless RML and 22L prions was assessed after 2 hours of PK digestion at 37°C. Graphs show the mean and SE from 4 mice per prion.

To characterize the biochemical properties of the anchorless strains, we measured the levels of soluble and insoluble PrP in the brains of mice infected with anchored and anchorless RML and 22L prions. For all samples tested, greater than 70% of the PrP was insoluble, consistent with previous reports [31], [32]. The anchorless prions showed a slightly higher ratio of insoluble : soluble PrP as compared to their anchored counterparts (GPI^−^RML: 93±1% versus RML: 73±5% ; GPI^−^22L: 89±2% versus 22L: 78±1%; Student's t-test p<0.05 for RML and 22L) (Figure 3A).

RML has been previously shown to be composed of primarily proteinase K (PK)-sensitive PrP^Sc^, with only approximately 20% PK-resistant PrP^Sc^ [33]. To investigate whether the GPI^−^RML shows a similar proportion of PK-resistant aggregates, equal volumes of PK-digested and non-digested brain homogenate samples were ultracentrifuged. The insoluble fractions were compared by western blotting, and revealed that anchored RML contained approximately 40–60% PK-resistant PrP, whereas the anchorless RML contained approximately 80–100% PK-resistant PrP (Figure 3B). Similar results were seen with the 22L and anchorless 22L prions (Figure 3B). PK-sensitivity was also tested by exposing brain homogenates to PK concentrations ranging from 0–9000 µg/ml for 2 hours at 37°C. The anchorless prions were significantly more PK-resistant than the anchored prions (Figure 3C).

### Anchorless prions show high stability in chaotropes

**Figure 4:**
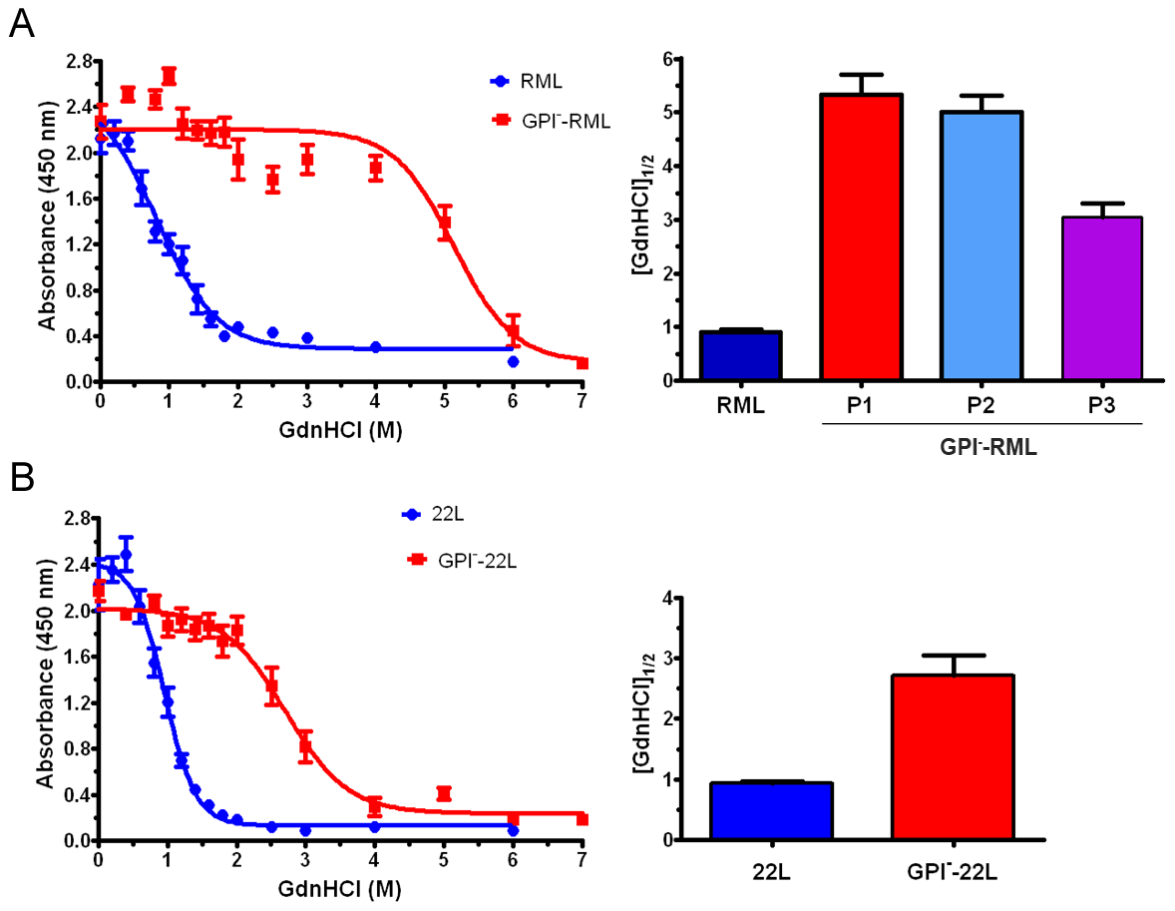
Conformational stability assay of GPI-anchored and anchorless prions. GPI-anchorless prions were significantly more resistant to GdnHCl-induced denaturation as compared to the GPI-anchored prions. (A) [GdnHCl_1/2_] of anchored and anchorless RML over three passages in the Tg(GPI^–^PrP) mice was measured by ELISA. (B) [GdnHCl_1/2_] of anchored and anchorless 22L. Plotted are the averages from n = 4 mice per prion, each run in triplicate. Graph (right panel) represents mean ± SE for all mice from [GdnHCl_1/2_] values that were independently calculated for each mouse.

Fibrillar prions were previously found to be more stable in chaotropes than non-fibrillar prions [26]. To determine the stability of the anchorless fibrillar prions, we performed standard conformational stability assays [34] and compared anchored and anchorless RML and 22L prions. We exposed brain homogenates to guanidine hydrochloride (GdnHCl) concentrations from 0 to 6 M, diluted the GdnHCl, digested samples with PK, quantified PrP by direct ELISA, and calculated the concentration at which half the PrP signal was lost, denoted [GdnHCl]_1/2_. Interestingly, lack of a GPI anchor resulted in the formation exceedingly stable prions, as the [GdnHCl]_1/2_ values were 5.3±0.4 for GPI^−^RML and 0.91±0.1 for RML (p<0.001) (Figure 4A). Additionally, the [GdnHCl]_1/2_ of anchorless RML decreased with serial passaging (second passage 5.0±0.30 and third passage 3.1±0.25 from n = 4 mice each) (Figure 4A). Similarly, GPI^−^22L showed a higher [GdnHCl]_1/2_ than 22L (GPI^−^22L: 2.7±0.3 and 22L: 0.82±0.1, p<0.001) (Figure 4B).

### Thermal denaturation of the anchorless prion strains

**Figure 5:**
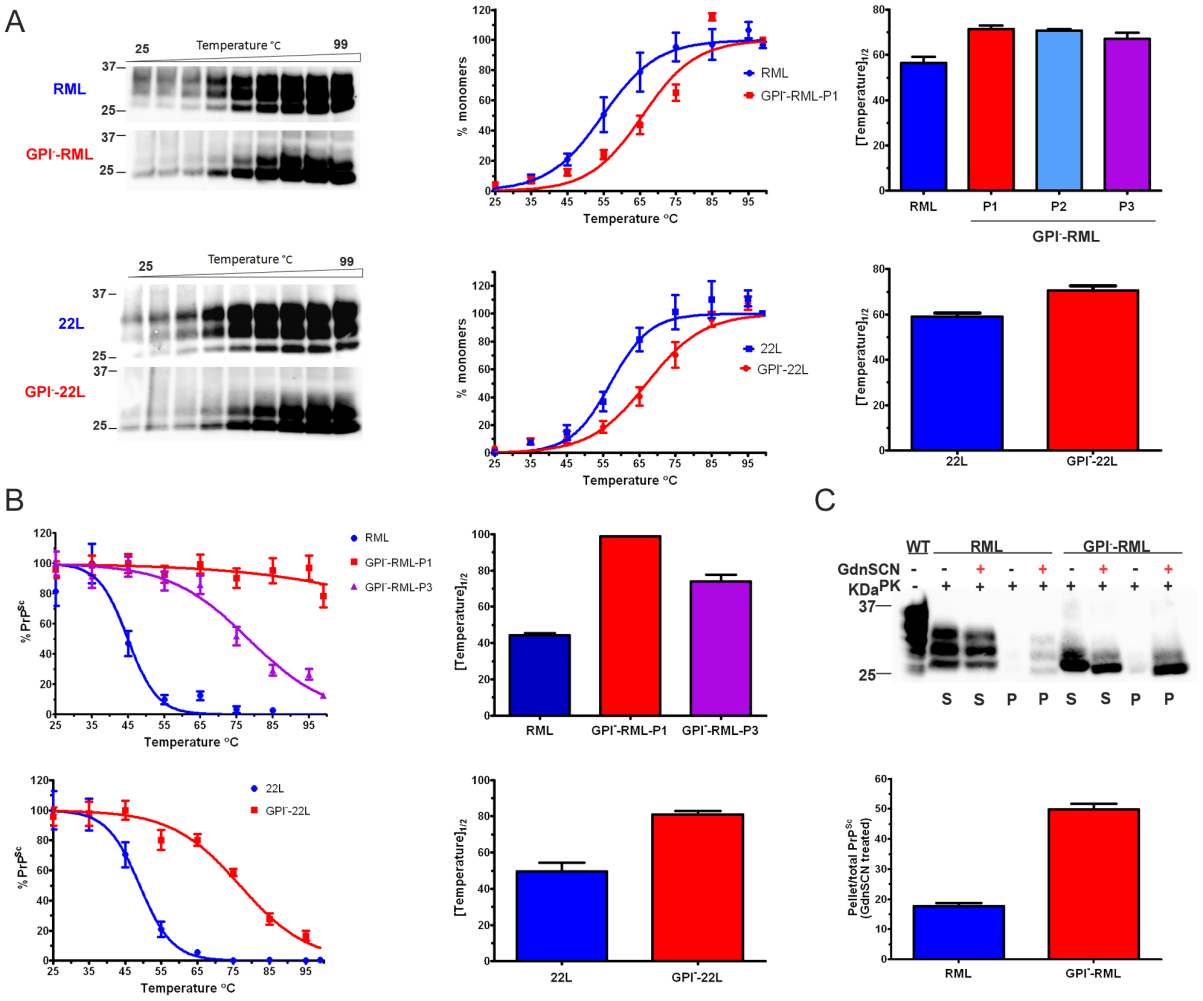
Thermal denaturation of anchored and anchorless prions. (A) PK-digested anchored and anchorless RML prions were heated to temperatures from 25–99°C and monomers were resolved by SDS-PAGE. Middle graphs show the plot of mean ± SE of RML and GPI^–^RML, passage 1 (P1) at each temperature (n = 4 mice each). The right graph shows the T_1/2_ point for RML and serial passages of GPI^–^RML. There were no significant differences among the passages of GPI^–^RML. Anchored and anchorless 22L were similarly assessed. (B) Samples were heated as in (A) and centrifuged. The remaining insoluble PrP^Sc^ was denatured and measured by ELISA. Plotted are the averages from n = 4 mice per prion, each run in triplicate. Data were normalized to the initial PrP^Sc^ intensity. Right panel, T_1/2_ values were independently calculated for each mouse and plotted (mean and SE). (C) Two aliquots of each of the anchored and anchorless prions were PK-digested, heated in SDS to 95°C and centrifuged, and the supernatants and pellets were collected. One aliquot of each supernatant (S) and pellet (P) was denatured with guanidine isothiocyanate (GdnSCN), and all samples were heated to 95°C prior to SDS-PAGE. Graph below shows mean ± SE from 4 independent experiments using 3 RML and 3 GPI^–^RML samples (RML: 18±1, GPI^–^RML: 50±2, p = 0.0001, Students t–test).

We sought to further assess the aggregate stability differences between anchored and anchorless prions using a thermal gradient. PK-digested aliquots of brain homogenate in SDS were heated to temperatures ranging from 25–99°C and proteins were immediately resolved by one dimensional denaturing gel electrophoresis. Monomeric PrP was quantified and plotted at each temperature. In comparing the temperature at which half of the PrP^Sc^ disassembled into monomers (T_1/2_), GPI^−^RML was found to be only modestly, but significantly more resistant to SDS thermal disassembly than RML (T_1/2_ of GPI^−^RML: 71±2°C and RML: 56±3°C, p<0.01) (Figure 5A). Similarly the T_1/2_ of the GPI^−^22L was significantly greater than 22L (GPI^−^22L: 71±2°C and 22L: 59±2°C, p<0.01) (Figure 5A).

The modest difference in thermal stability between anchorless and anchored strains was surprising, yet an underlying assumption for the thermal stability assays is the complete denaturation of PrP^Sc^ at 99°C for SDS-PAGE. To exclude the possibility of higher order aggregates not migrating through the gel, we measured the insoluble PrP^Sc^ by ELISA. Brain homogenate samples were heated in SDS to temperatures from 25 to 99°C and centrifuged. The insoluble pellet fractions were denatured using 6 M guanidine isothiocyanate and PrP^Sc^ levels were then measured by ELISA. RML showed low levels of insoluble PrP at low temperatures which sharply decreased to nearly zero at approximately 55°C (T_1/2_ of 44±1°C). In stark contrast, the GPI^−^RML (first passage) showed abundant insoluble PrP with no loss of signal, even at the highest temperatures (Figure 5B), indicating that there was substantial PrP^Sc^ that remained insoluble, even after heating to 99°C in SDS. Interestingly, in the third passage GPI^−^RML, PrP^Sc^ became largely soluble at high temperatures (T_1/2_ of 74±4°C), indicating that the thermostability decreased with passaging, consistent with results from the GdnHCl denaturation experiments. Similar to RML, the anchored 22L was also significantly less thermostable than the GPI^−^22L [T_1/2_ of 50±5°C (22L) versus 81±2°C (GPI^−^22L); p<0.01], indicating that abundant GPI-anchorless PrP^Sc^ remained insoluble at high temperatures (Figure 5B).

We reasoned that if the low thermostability values in the initial experiments (analyzed by western blot) were due higher order aggregates, these larger aggregates should migrate after complete denaturation. We PK-digested, heated to 95°C SDS, and centrifuged two aliquots each of anchored and anchorless RML. One aliquot of each supernatant and pellet fraction was denatured with 6 M guanidine isothiocyanate while the other was not treated, and then all samples were heated to 95°C in SDS prior to SDS-PAGE. As expected, the soluble anchored and anchorless PrP^Sc^ signals in the supernatant fractions were not altered following guanidine isothiocyanate denaturation. In contrast, for the pellet fractions, there was a slight enhancement of the RML signal and a massive increase in the anchorless RML signal with denaturation (Figure 5C, last 2 lanes). In sum, the insoluble fraction of GPI^−^RML remained highly aggregated after heating in SDS, requiring the additional denaturation with a strong chaotrope to fully disassemble aggregates into monomers for electrophoresis. This result confirms that heating the GPI^−^RML in SDS disassembles only half the population of the total anchorless PrP^Sc^ and this property likely underlies the modest differences in thermal stability initially observed by SDS-PAGE. Thus the denaturation required for the ELISA enables a much more accurate measurement of the total fibrillar prion population.

### GPI^−^RML infection of WT mice

**Figure 6:**
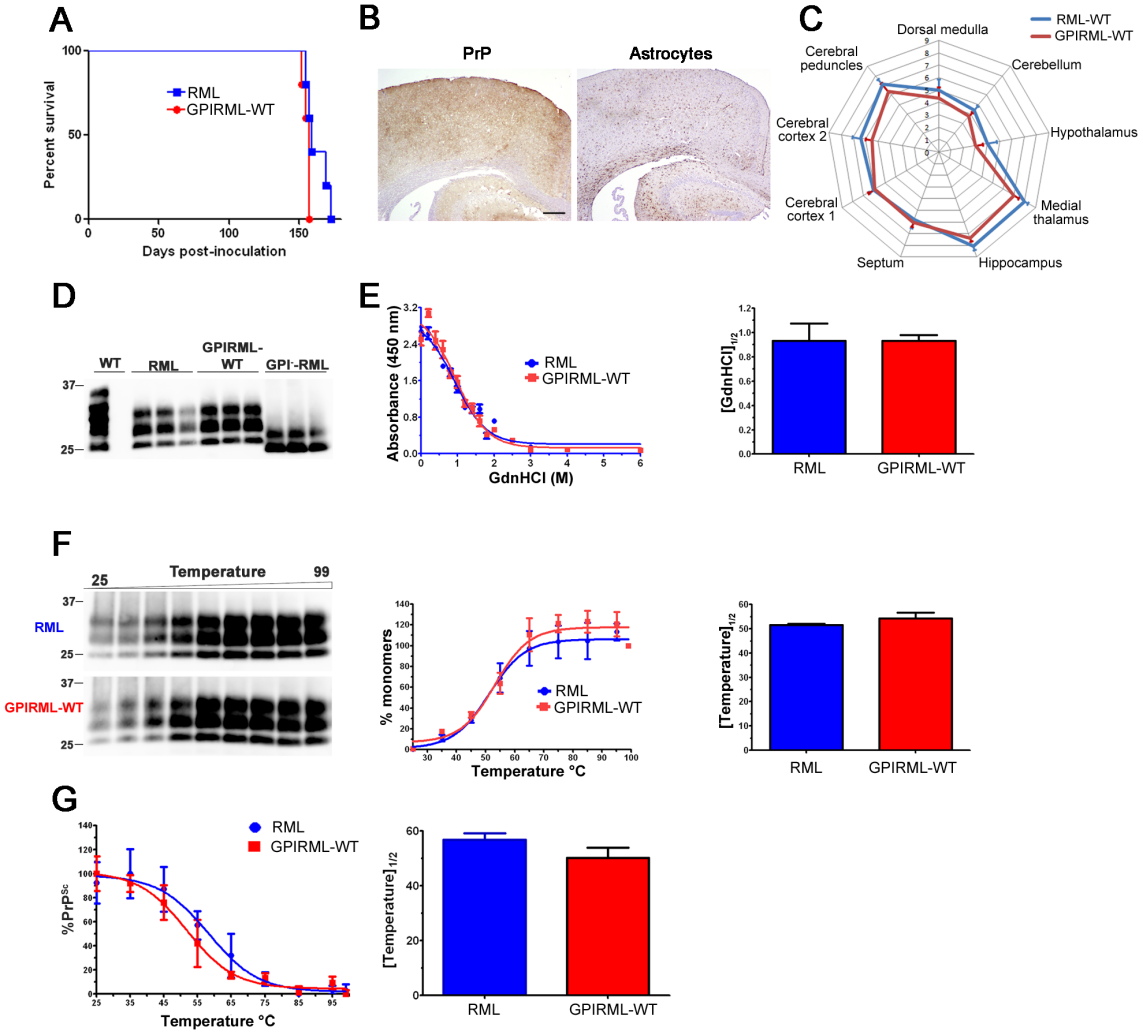
GPI^–^RML prions inoculated into WT mice (GPIRML-WT). (A) Survival curves of WT mice inoculated with RML or GPI^–^RML prions (passage 2). (B) PrP and astrocyte immunostaining of brain sections at the level of cerebral cortex and hippocampus show diffuse PrP^Sc^ deposits and accompanying gliosis. (C) Lesion profile analysis. For RML- and GPIRML-infected WT mice, the severity of spongiosis, astrogliosis, and PrP^Sc^ deposition were scored for nine brain regions (see Methods) and were nearly superimposable. Each ring represents 1 point. (D) PK-digested brain samples from WT mice infected with RML or GPI^–^RML show no difference in their glycoform profiles. Also shown are Tg(GPI^–^RML) mice infected with RML, which show a shift to a lower molecular weight and only mono-glycosylated PrP. (E) Conformational stability and (F) thermal stability of RML and GPIRML-WT were nearly identical (n = 4 mice each). (G) Thermal denaturation curves of RML- and GPIRML-infected WT mice show the insoluble PrP^Sc^ remaining after heating to various temperatures as measured by ELISA. Right panel, T_1/2_ values were independently calculated for each mouse and plotted. For (E), plotted are the averages from n = 4 mice per prion, each run in triplicate. For (F), the middle graph shows the mean ± SE of RML and GPI^–^RML (n = 4 mice each). Graphs (right panels) represent the mean ± SE for all mice. Scale bar = 500 µm.

To determine whether the anchorless RML would revert back to an RML disease phenotype in WT mice, we intracerebrally inoculated anchorless RML prions into WT mice. The anchorless RML prions induced disease in WT mice at a time period similar to RML prions (Figure 6A). The PrP^Sc^ aggregate morphology was diffuse and widespread in the cerebral cortex, basal ganglia thalamus, hippocampus, cerebellum, and brainstem (Figure 6B), and there were no differences in the distribution and degree of the spongiosis, astrogliosis, or PrP^Sc^ as seen by a lesion profile analysis (Figure 6C). RML and GPIRML-WT also showed indistinguishable glycoform profiles (Figure 6D, NSD between quantified glycoform ratios), stability in chaotropes ([GdnHCl]_1/2_ for RML: 0.93 and GPIRML-WT: 0.93) (Figure 6E) and thermostability (Figure 6F, G) [Fig. 6F: T_1/2_: 51±1°C (RML) and 54±3°C (GPIRML-WT)]. These data suggest anchorless RML prions are efficiently transmitted to mice expressing GPI-anchored PrP.

### Human full length anchorless prions from a patient are also exceedingly stable in denaturants

**Figure 7:**
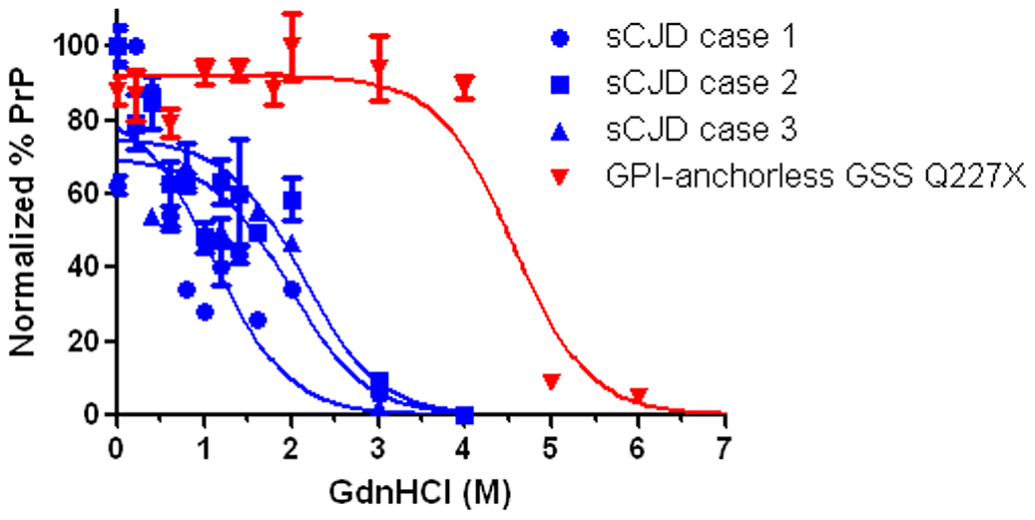
Conformational stability of anchorless prions from a human patient. GPI-anchorless human prions were more stable in GdnHCl than GPI-anchored prions from 3 patients with sporadic Creutzfeldt-Jakob disease (sCJD), type MM1 ([GdnHCl_1/2_] for GPI-anchorless case: 4.6 ; for sCJD cases: 1.1, 2.0, 2.1).

To determine whether the high chaotrope stability of anchorless prions in mice is also seen with anchorless prions in patients, we tested brain from a patient expressing full length anchorless PrP due to a very rare Q227X mutation. This patient died at 45 years of age and the brain showed extensive plaque-like prion deposits and a lack of spongiosis. Although full length PrP was expressed, the PK-resistant core fragment was primarily a 7 kD band that lacked the amino and carboxy termini [35]. We measured the chaotrope stability of anchorless prions and found that the [GdnHCl]_1/2_ was markedly higher for the anchorless prions than for three sporadic CJD (MM1) cases (Figure 7).

## Discussion

Prions exhibit a tremendous conformational repertoire, yet the relationship between their misfolded structure and disease phenotype is unclear. We have previously found that fibrillar prions are inefficient at trafficking to the CNS and are highly stable under denaturing conditions [26]. In the present study, we perform a comprehensive analysis of the biochemical properties of a prion in its anchored and anchorless state and correlate results with the disease pathogenesis. Previous studies have focused on the first passage of prions in Tg(GPI^–^PrP) mice. Baron and colleagues showed that anchored and anchorless 22L exhibit a similar secondary structure with residues 81–167 highly protected against deuterium incorporation [31]. Here we demonstrate that serially passaged anchorless prions have a substantial resistance to denaturation, a fibrillar morphology, and are poorly neuroinvasive, thus differ markedly from their nonfibrillar anchored counterpart. Additionally, anchorless prions show a high ratio of insoluble to soluble PrP, suggestive of efficient conversion. Thus there is a profound biochemical transformation when a nonfibrillar prion is converted by extracellular anchorless PrP^C^.

We observed that anchorless prions were exceedingly stable when exposed to chaotropes and heat. Of note, even heating anchorless prions to 95°C in SDS solubilized only half the PrP^Sc^ population into monomers for SDS-PAGE analysis. Our previous analysis revealed that fibrillar anchored prions were also highly stable, reinforcing the view that fibrillar prions in general may be more stable in chaotropes than many nonfibrillar prions. What may be the structural underpinnings of the high stability of a fibrillar prion? Within fibrils, β-sheets are arranged perpendicular to the fibril axis, with H-bonds forming a dry interface between β-strands [36], [37], [38], [39]. The observation that the anchorless prions are stable in chaotropes raises the possibility of long β-sheets, more H-bonds, or increased strength of H-bonds between β-strands. Indeed, H-bond strength can differ among prion fibrils [40]. However, length of the β-sheet core can also differ. An elegant study of two yeast prion strains revealed a longer stretch of amino acids constituting the amyloid core in the more stable strain [41], [42]. Thus far in mammalian prions, H/D exchange studies of anchorless versus anchored prions have revealed only small differences in the length of the buried core, which is suspected to be β-sheeted [43].

Intriguingly, anchorless prions accumulate to very high levels over a relatively short incubation period in Tg(GPI^–^PrP) mice, a finding also noted in the passage of spontaneous anchorless prions [22]. This differed from findings observed in other fibrillar prions, which tend to have low levels of PrP^Sc^ and very long incubation periods [26], [27], [44]. What mechanism may underlie the relatively short incubation period? Anchorless prions may rapidly convert monomers due to increased interactions between extracellular PrP^Sc^ and secreted PrP^C^. Alternatively, the extracellular location may promote interactions with co-factors that enhance conversion of anchorless PrP [45]. Degradation and clearance of anchorless prion fibrils may be exceedingly poor, as suggested by their ability to resist high concentrations of PK. Lastly, it is possible that there is a high frangibility leading to release of many small aggregate “seeds” and subsequent efficient recruitment of PrP^C^. Although the latter may seem unlikely from the high stability measurements, it remains possible since frangibility was not directly measured.

Anchorless RML prions readily switched back into a nonfibrillar form when inoculated intracerebrally into WT mice (IP inoculations are ongoing). Pathological features resembled RML and were characterized by diffuse PrP^Sc^ deposits, extensive spongiosis and astrogliosis. The lesion profiles, incubation period after IC inoculation, stability in chaotropes and at high temperatures, and glycoform profiles were indistinguishable from RML. Nevertheless, Mahal and colleagues showed interesting differences between the RML and anchorless RML passaged into WT mice using the Extended Cell Panel Assay, indicating that anchorless RML passaged in WT mice produces a novel strain [46].

Similar to the inefficient neuroinvasion we observed with the anchorless RML prions in Tg(GPI^–^PrP) mice, the neuroinvasion of WT-RML prions was also infrequent in Tg(GPI^–^PrP) mice in an extensive study in which mice were inoculated by intravenous, intraperitoneal, intraocular, intranerval, and intralingual routes [25]. After intralingual inoculation, no prions were detected in the CNS, indicating a robust barrier to neuroinvasion despite the limited distance for prion transport from tongue to brain. Even after direct intranerval (sciatic) inoculation, prions were not detected in the brain, however prions accumulated in the lumbar spinal cord ipsilateral to the injection site, indicating very slow conversion along the injected nerve [25]. In the present study, serially-passaged, more rapidly lethal anchorless prions were only weakly neuroinvasive in Tg(GPI^–^PrP) mice, indicating that the adapted prions remained slow to invade the CNS, although prions readily accumulated at extracerebral sites, including the spleen, adipose tissue, and heart. These findings are reminiscent of other fibrillar prions that are poorly neuroinvasive, including 87 V [26], [27], mCWD [26], and vCJD in transgenic mice expressing human PrP [28], and may indicate that the common fibrillar structure effectively impairs a prion's ability to neuroinvade. Although 87 V and mCWD are also highly stable, stability does not seem to correlate with neuroinvasion, as some highly stable strains can efficiently neuroinvade [16]. Instead only the fibrillar structure correlates with inefficient neuroinvasion ability.

In conclusion, nonfibrillar and fibrillar anchored prion strains all converged into fibrillar prions in mice expressing anchorless PrP, and GPI^–^RML reverted back to a nonfibrillar form in mice expressing anchored PrP. To earlier evidence that anchorless prions are poorly neuroinvasive, fibrillar, and exhibit a new tropism for adipose tissue and heart, here we add that anchorless prions are exceedingly stable in denaturants, resist enzyme degradation, and even after serial passage, remain inefficient at neuroinvasion. These features are common to anchored fibrillar prions and suggest that having a fibrillar structure hinders invasion of the CNS from extraneural sites. Therefore other anchored or anchorless fibrillar prions that accumulate in non-neural tissues would be expected to show very slow spread to the brain or spinal cord.

## Materials and Methods

### Ethics statement

All procedures involving animals were performed to minimize suffering and were approved by the Institutional Animal Care and Use Committee at UC San Diego. Protocols were performed in strict accordance with good animal practices, as described in the *Guide for the Use and Care of Laboratory Animals* published by the National Institutes of Health.

### Prion inoculations

WT (C57BL/6) or Tg(GPI^−^PrP) mice (groups of n = 4–5 mice) were inoculated IC into the left parietal cortex with 30 µl or inoculated IP with 100 µl of a 0.1% (w/v) prion-infected brain homogenate prepared from terminally ill mice. Mice were monitored three times weekly, and TSE was diagnosed according to clinical criteria including ataxia, kyphosis, stiff tail, hind leg clasp, and hind leg paresis. Mice were sacrificed at the onset of terminal disease and incubation period was calculated from the day of inoculation to the day of terminal clinical disease. Mice were maintained under specific pathogen-free conditions. All procedures involving animals were performed to minimize suffering and were approved by the Institutional Animal Care and Use Committee at UC San Diego. Protocols were performed in strict accordance with good animal practices, as described in the Guide for the Use and Care of Laboratory Animals published by the National Institutes of Health.

### Histopathology and immunohistochemical stains

Two-µm thick sections were cut onto positively charged silanized glass slides and stained with hematoxylin and eosin, or immunostained using antibodies for PrP (SAF84) or astrocytes (GFAP). For PrP staining, sections were deparaffinized and incubated for 5 min in 88% formic acid and treated with 5 µg/ml of proteinase-K to expose epitopes. Sections were then autoclaved in citrate buffer (pH 6). Immunohistochemical stains were performed using the TSA Plus DNP kit (PerkinElmer). Sections were blocked and incubated with anti-PrP SAF-84 (SPI bio; 1∶400) for 45 min followed by anti-mouse HRP (Jackson Immunolabs; 1∶500) for 30 min. Slides were then incubated with anti-DNP-HRP (PerkinElmer, 1∶100) for 30 min, followed by 6 min incubation with DAB. Sections were counterstained with hematoxylin. GFAP immunohistochemistry for astrocytes (1∶500; DAKO) was similarly performed, however with antigen retrieval by PK-digestion (20 ug/ml for 10 min at room temperature).

### Lesion profile

We selected 9 anatomic brain regions in accordance with previous strain-typing protocols from 5 mice per group [47], [48]. We scored spongiosis, gliosis, and PrP immunological reactivity on a scale of 0–3 (not detectable, mild, moderate, and severe). A sum of the three scores resulted in the value obtained for the lesion profile for the individual animal. The ‘radar plots’ depict the scores for spongiform changes, gliosis and PrP deposition. The following brain regions were scored: dorsal medulla, cerebellum, hypothalamus, medial thalamus, hippocampus, septum, medial cerebral cortex dorsal to hippocampus, medial cerebral cortex dorsal to septum, white matter at cerebral peduncles. An investigator blinded to animal identification performed the histological analyses.

### Western blotting for PrP^Sc^ in brain and spleen

For brain and spleen from the IP-inoculated mice, sodium phosphotungstic acid (NaPTA) PrP^Sc^ precipitation was performed as previously described [49]. Briefly, brain and spleen extracts in PBS containing 2% sarkosyl were digested with an endonuclease [Benzonase (Sigma)] followed by treatment with 50 µg/ml PK at 37°C for 30 min. After addition of NaPTA, MgCl_2_, and protease inhibitors, extracts were incubated at 37°C for 30 min, and centrifuged at 18,000 g for 30 min at 37°C. Pellets were resuspended in 0.1% sarcosyl for electrophoresis and blotting. For IC-inoculated mice, brain extracts in lysis buffer (10 mM Tris-HCl pH 7.4, 150 mM NaCl, 2% sarcosyl) were digested with 50 µg/ml PK at 37°C for 40 min.

Samples were electrophoresed through 10% Bis-Tris gels (Invitrogen) and blotted onto a nitrocellulose membrane. PrP was detected using monoclonal antibody POM1 (epitope in the globular domain, amino acids 121–231, a kind gift from Dr. Adriano Aguzzi) [50] and an HRP-conjugated anti-mouse IgG secondary antibody. The blots were developed using a chemiluminescent substrate (ECL detection Kit, Pierce) and visualized on a Fuji LAS 4000 imager. Quantification of PrP^Sc^ glycoforms was performed using Multigauge V3 software (Fujifilm).

### Conformation stability assay

Prion strain stability in GdnHCl was performed as previously described [34] with minor modifications. Briefly, brain homogenates were denatured in GdnHCl ranging from 0–6 M for 1 hr and then diluted to 0.15 M GdnHCl. The samples were then digested with PK at a ratio of 1∶500 (1 µg PK : 500 µg total protein) for 1 hr at 37°C, stopped with PMSF and Complete protease inhibitor (Roche), and centrifuged at 18,000 g for 1 hr. The pellets were washed with 500 µl of 0.1 M NaHCO_3_ (pH 9.6) and centrifuged for 20 min at 18,000 g. Pellets were denatured in 6 M guanidine isothiocyanate (GdnSCN), diluted 2X with 0.1 M NaHCO_3_, and coated passively onto an ELISA plate. PrP was detected with anti-PrP biotinylated-POM1 antibody, a streptavidin HRP-conjugated anti-mouse IgG secondary antibody, and detected with a chemiluminescent substrate. Each strain was analyzed in at least 3 separate experiments using 4 mice. Statistical analysis was performed using a Student's *t* test. The human brain samples were detected using the anti-PrP 3F4 antibody, a biotinylated anti-mouse secondary antibody, and streptavidin-HRP followed by the chemiluminescent substrate.

### Thermal denaturation assay

Brain homogenate in a Tris lysis buffer (10 mM Tris-HCl pH 7.4, 150 mM NaCl, 2% sarcosyl) was digested with 50 µg/ml PK for 40 min at 37°C. PK digestion was inactivated with phenylmethylsulfonyl fluoride (PMSF) (2 mM final concentration) and Complete protease inhibitor. Aliquots were incubated in 1.6% SDS (final) and heated to temperatures ranging from 25°C to 99°C (10° intervals) for 6 min with shaking in a thermomixer at 1000 rpm. Western blotting was performed and PrP signals from monomers were captured and quantified using a Fujifilm LAS-4000 imager and Multigage software. Each strain was analyzed in at least 3 separate experiments using 4–11 mice.

### Quantification of soluble and insoluble PrP

Brain homogenate in a Tris lysis buffer was maintained at 37°C for 15 min, centrifuged at 150,000 g for 1 hr at 4°C, and separated into supernatant and pellet fractions. Proteins in the supernatant were precipitated using cold methanol. Supernatant and pellet proteins were then analyzed and quantified by western blotting for PrP. Each strain was analyzed in at least 3 separate experiments using 3–4 mice.

### Quantification of PK resistant and sensitive PrP

Brain homogenate in a Tris lysis buffer was maintained at 37°C for 15 min and split into two aliquots. One aliquot was treated with PK (50 µg/ml) at 37°C for 30 min, and both aliquots were centrifuged at 150,000 g for 1 hr at 4°C. PrP in the PK-treated and untreated pellets were quantified by western blotting. Each strain was analyzed in at least 3 separate experiments using 4 mice.

### Quantification of PK-resistant PrP by ELISA

Equal amount of sample in a Tris lysis buffer was maintained at 37°C for 15 min, digested with PK (50 µg/ml) at 37°C for 40 min, and the PK was inactivated with PMSF (6 mM final). The samples were denatured with GndHCl (2 M final) at 81°C for 6 min, diluted 10X with 0.1% TBST and added to an ELISA plate precoated with POM-2 antibody. PrP was detected using anti-PrP biotinylated-POM1 antibody, a streptavidin HRP-conjugated anti-mouse IgG secondary antibody, and 1-Step Ultra TMB-ELISA substrate (Thermo-Scientific). Each strain was analyzed in triplicate using 4 mice each. Statistical analysis was performed using a Student's *t* test.

### Electron microscopy

Tissues were post-fixed in osmium tetroxide, embedded in epon araldite, sectioned with the ultramicrotome, then collected on grids and post-stained using saturated uranyl acetate solution and bismuth sub-nitrate. Grids were analyzed with a Zeiss EM10 electron microscope.

### PTAA staining of frozen tissue sections

Frozen sections from mouse brain were dried for 1 hour and fixed in 100% and 70% ethanol for 10 min each. After washing with deionized water, sections were equilibrated in 100 mM sodium carbonate at pH 10.2 for 30 minutes. The PTAA was diluted in the sodium carbonate buffer (1 µg : 100 µl buffer) and added to the sections. The sections were incubated with PTAA for 30 min at room temperature and washed with sodium carbonate buffer. The spectra in the tissue were recorded with a Leica DM6000 B fluorescence microscope (Leica Microsystems, Wetzlar, Germany) fitted with a Spectraview 4.0 (Applied Spectral Imaging, Migdal, Israel) and a Spectra-Cube (interferometrical optical head SD 300) module with cooled CCD-camera, through a 436/10 nm (LP 475) bandpass filter in steps of 6 nm. The data were processed with SpectraView 3.0 EXPO. Spectra were collected from 8 individual spots within 3–5 plaques from a minimum of two different cases of each prion-infected brain.

### Human patient samples

The human brain samples were from three patients diagnosed with sCJD, consisting of one male (age 63) and two females (ages 53 and 60). All had a *PRNP* genotype encoding 129 MM PrP, a short disease duration (2–3 months), a type 1 PrP^Sc^ pattern on western blot, and histopathology typical of classic sCJDMM1. Case typing was performed at the National Prion Disease Pathology Surveillance Center at Case Western Reserve University in Cleveland, Ohio. The GPI-anchorless Q227X GSS-type prion case was identified at the Dutch Surveillance Centre for Prion Diseases, University Medical Centre Utrecht in The Netherlands.

## Supporting Information

Figure S1 **PrP ELISA measurements of PK-digested PrP^Sc^.** First through third passages (P1, P2, and P3) of GPI^–^RML in Tg(GPI^–^PrP) mice were assessed (n = 4 mice each). Graph shows mean ± SE. Passage 1 was significantly different than passage 2 (Student's t-test, p<0.01).
(TIF)

Figure S2 **Ultrastructure of brain (cerebral cortex) from a Tg(GPI^–^PrP) mouse infected with GPI^–^RML prions.** (A) Low power image of a blood vessel (V) surrounded by extracellular, loosely arranged fibrils (*). (B) High power image shows fibrils are short and present in thin bundles that are haphazardly arranged. (C) Commonly seen were dystrophic neurites (arrows) containing variably-sized electron dense deposits.
(TIF)

Figure S3 **Immunohistochemical stains of heart and adipose tissue (brown fat) for PrP.** PrP^Sc^ deposits were observed in heart and adipose tissue of Tg(GPI^–^PrP) mice for all strains tested. Scale bars = 100 µm.
(TIF)

Figure S4 **PrP^Sc^ in brains and spleens of Tg(GPI^–^PrP) mice inoculated with anchorless RML prions.** Brain from only one of five mice showed detectable PrP^Sc^ by (A) NaPTA precipitation and western blot, or (B) ELISA. (C) In contrast, all spleens from GPI^–^RML inoculated Tg(GPI^–^PrP) mice as well as RML-inoculated WT mice showed PrP^Sc^ detectable by NaPTA precipitation and western blot. Approximately 7-fold more total protein was loaded for WT as compared to the Tg(GPI^−^PrP) spleen.
(TIF)
