# Supplementary figures and images for: Defining the Conformational Features of Anchorless, Poorly Neuroinvasive Prions
Source: PLoS Pathog. 2013 Apr 18;9(4):e1003280. doi: 10.1371/journal.ppat.1003280 (PMC3630170; doi:10.1371/journal.ppat.1003280)

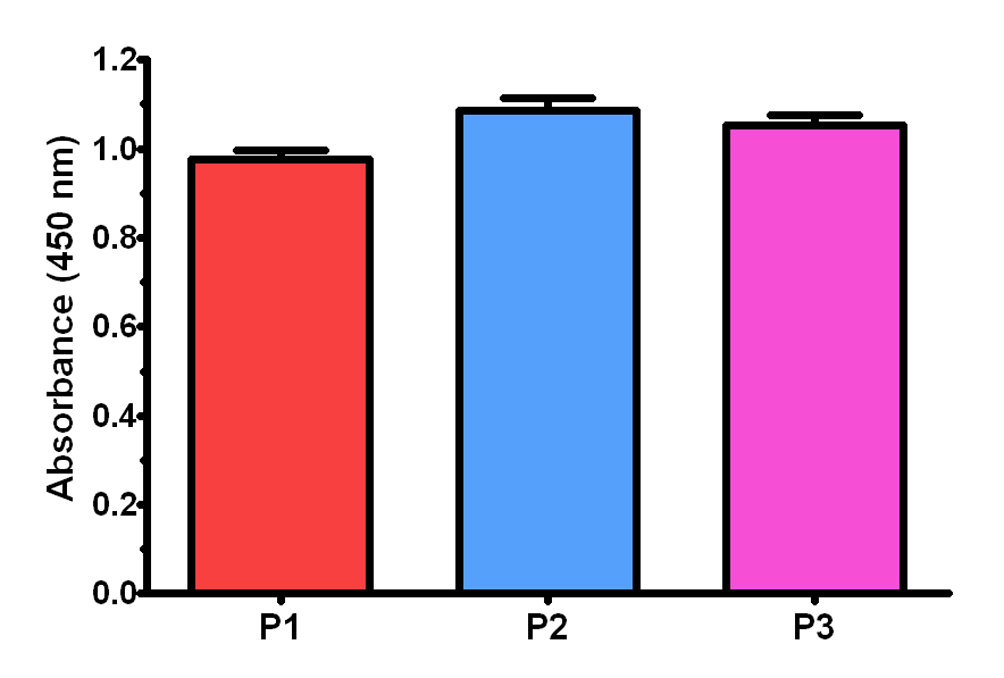

Supplement: Figure S1 — PrP ELISA measurements of PK-digested PrPSc. First through third passages (P1, P2, and P3) of GPI–RML in Tg(GPI–PrP) mice were assessed (n = 4 mice each). Graph shows mean ± SE. Passage 1 was significantly different than passage 2 (Student's t-test, p<0.01). (TIF) [file ppat.1003280.s001.tif]

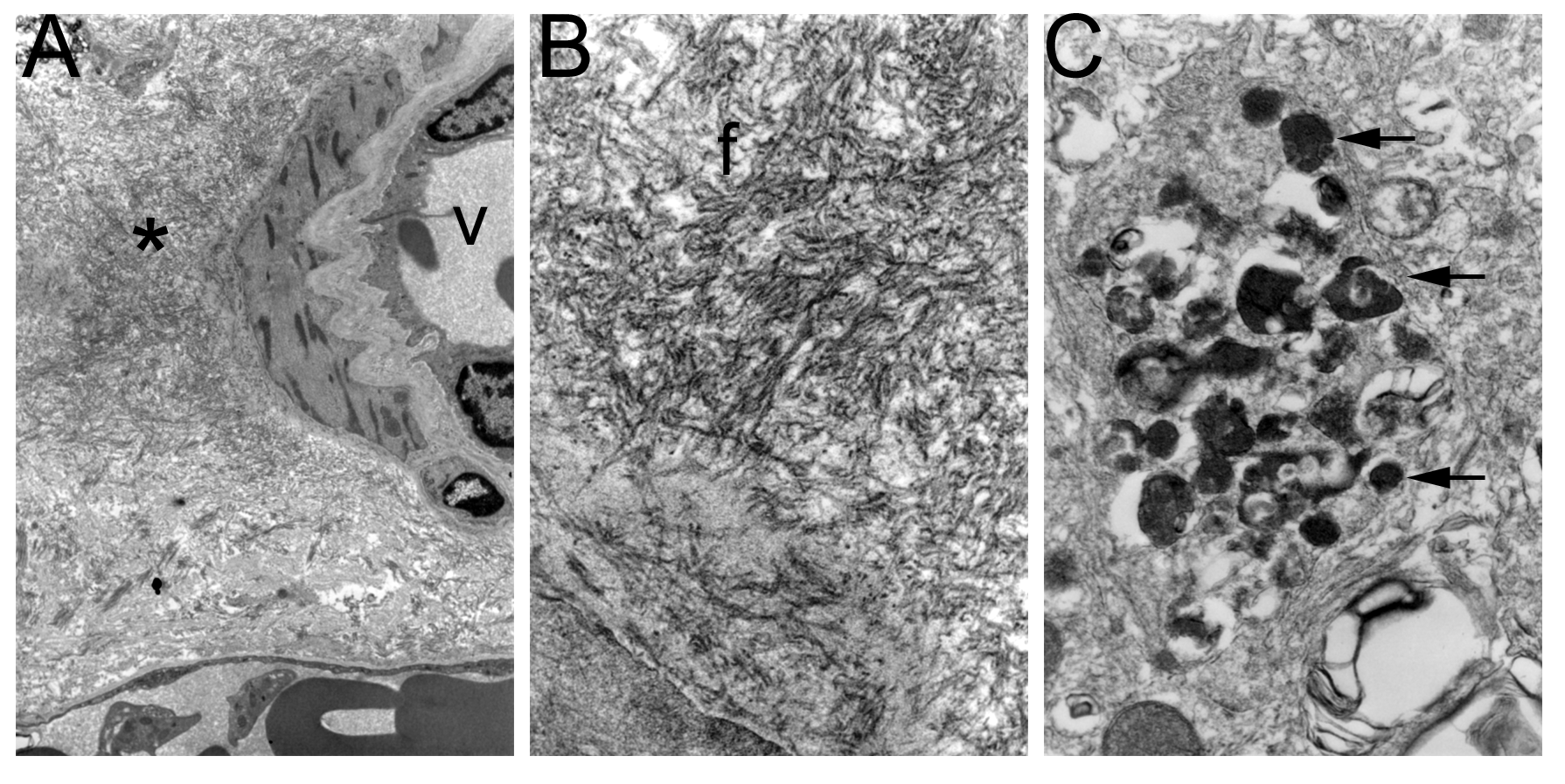

Supplement: Figure S2 — Ultrastructure of brain (cerebral cortex) from a Tg(GPI–PrP) mouse infected with GPI–RML prions. (A) Low power image of a blood vessel (V) surrounded by extracellular, loosely arranged fibrils (*). (B) High power image shows fibrils are short and present in thin bundles that are haphazardly arranged. (C) Commonly seen were dystrophic neurites (arrows) containing variably-sized electron dense deposits. (TIF) [file ppat.1003280.s002.tif]

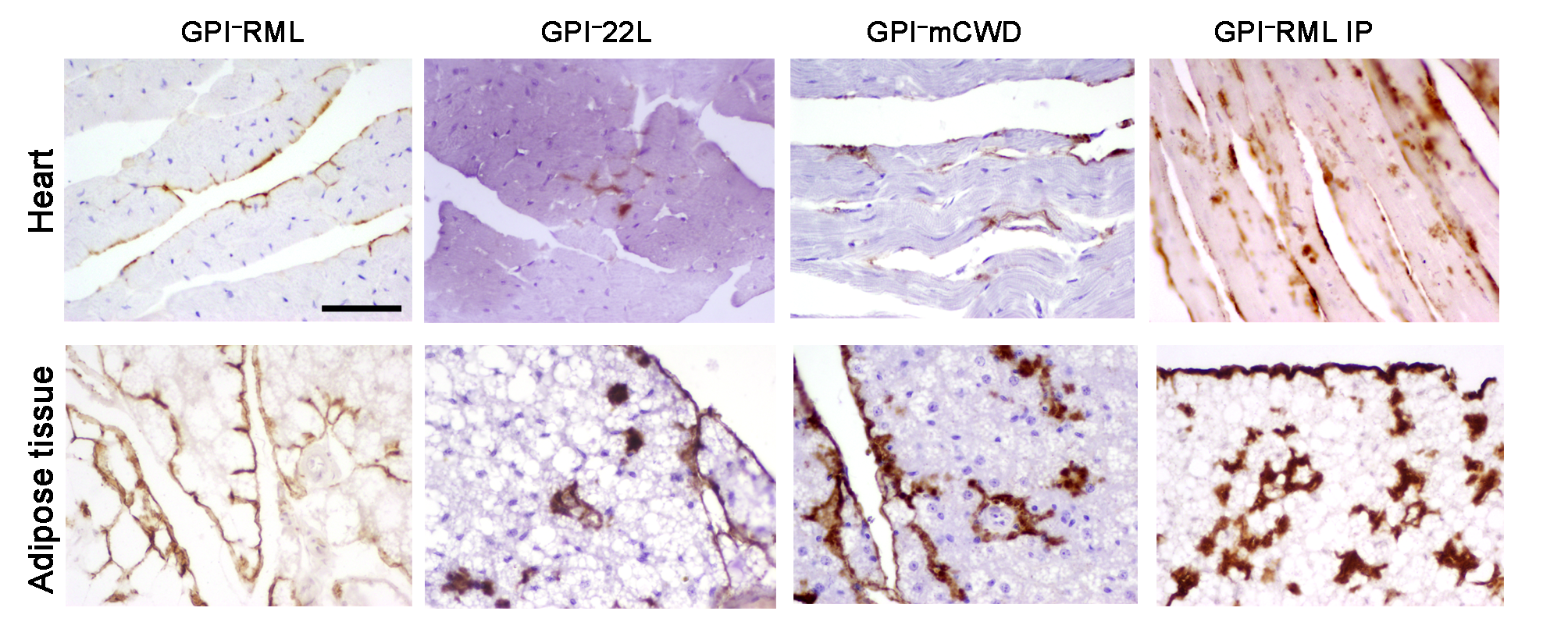

Supplement: Figure S3 — Immunohistochemical stains of heart and adipose tissue (brown fat) for PrP. PrPSc deposits were observed in heart and adipose tissue of Tg(GPI–PrP) mice for all strains tested. Scale bars = 100 µm. (TIF) [file ppat.1003280.s003.tif]

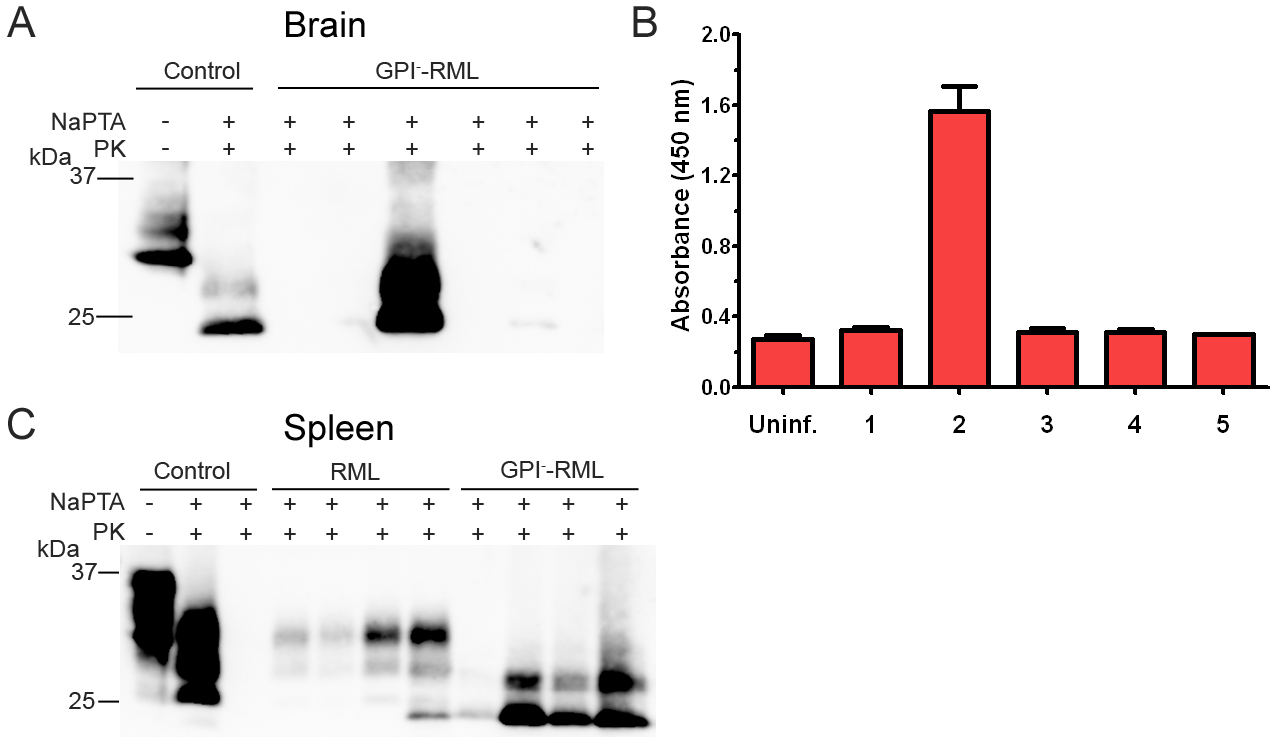

Supplement: Figure S4 — PrPSc in brains and spleens of Tg(GPI–PrP) mice inoculated with anchorless RML prions. Brain from only one of five mice showed detectable PrPSc by (A) NaPTA precipitation and western blot, or (B) ELISA. (C) In contrast, all spleens from GPI–RML inoculated Tg(GPI–PrP) mice as well as RML-inoculated WT mice showed PrPSc detectable by NaPTA precipitation and western blot. Approximately 7-fold more total protein was loaded for WT as compared to the Tg(GPI−PrP) spleen. (TIF) [file ppat.1003280.s004.tif]
